# Persistence in eye movement during visual search

**DOI:** 10.1038/srep20815

**Published:** 2016-02-11

**Authors:** Tatiana A. Amor, Saulo D. S. Reis, Daniel Campos, Hans J. Herrmann, José S. Andrade

**Affiliations:** 1 Computational Physics IfB, ETH Zurich, Stefano-Franscini-Platz 3, CH-8093, Zurich, Switzerland; 2 Departamento de Física, Universidade Federal do Ceará, 60451-970, Fortaleza, Ceará, Brazil; 3 Departament de Física, Universitat Autònoma de Barcelona, 08193, Bellaterra, Barcelona, Spain

## Abstract

As any cognitive task, visual search involves a number of underlying processes that cannot be directly observed and measured. In this way, the movement of the eyes certainly represents the most explicit and closest connection we can get to the inner mechanisms governing this cognitive activity. Here we show that the process of eye movement during visual search, consisting of sequences of fixations intercalated by saccades, exhibits distinctive persistent behaviors. Initially, by focusing on saccadic directions and intersaccadic angles, we disclose that the probability distributions of these measures show a clear preference of participants towards a reading-like mechanism (geometrical persistence), whose features and potential advantages for searching/foraging are discussed. We then perform a *Multifractal Detrended Fluctuation Analysis* (MF-DFA) over the time series of jump magnitudes in the eye trajectory and find that it exhibits a typical multifractal behavior arising from the sequential combination of saccades and fixations. By inspecting the time series composed of only fixational movements, our results reveal instead a monofractal behavior with a Hurst exponent 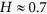, which indicates the presence of long-range power-law positive correlations (statistical persistence). We expect that our methodological approach can be adopted as a way to understand persistence and strategy-planning during visual search.

Eye-movement data provides information on the cognitive process underlying visual search^1^ and, in particular, visual search tasks can be used to shed some light on how the human (or animal) eye implements information for foraging and/or planning^2^,^3^,^4^. A number of studies have been conducted on the visual search of hidden objects in complex scenes^5^,^6^,^7^, going from cases of targets embedded in a noisy background^8^ to more simplified visual tasks on macaques^9^. Visual search, however, remains as a complex cognitive task which involves a number of diverse underlying mechanisms that need to be addressed. One of these essential mechanisms, whose properties are still under debate, is memory.

The first theories on visual search assumed the existence of visual memory in terms of an inhibitory mechanism that prevents an item to be revisited after identifying it as a distractor^10^, or in the capacity of collecting visual information in parallel for each item inspected^11^. Later, it was suggested that visual search involves a memoryless search strategy^12^. By studying the response time of a visual task involving the search of a target along with distractors, it was found that the efficiency of search remains constant across trials. This would imply that the visual system does not accumulate information about an item over time during a search episode and that subjects may frequently reinspect locations that have already been inspected. However, although subjects tend to reexamine some items, the pattern of revisitations does not match the one predicted by a memoryless model^13^. Thus, it has been argued that revisitations occur not because the searcher forgets the items that have been examined, but because the items were inadequately examined at first. So, while memory may not be a primary mechanism for instantaneous object recognition (in these scenarios saliency could be, for example, more significant^14^,^15^) it seems clear that it must play a role in cognition and, in particular, in strategy planning. Along these lines, the problem of visual memory has been recently addressed as a synonym of persistence for natural scenes in a number of works^16^,^17^,^18^. From the study of how subjects can recall unrelated items, removing semantic cues, it has been shown that visual memory over time performs better than had been thought. Therefore, persistence may represent a convenient conceptual measure to analyze the performance and/or efficiency of living beings performing real-world visual tasks.

In the aforementioned studies, persistence and memory were investigated through the analysis of purely cognitive measurements, such as the response time of subjects or the amount of objects recalled after inspecting a scene. One should note that in any visual task we are constantly gathering information from the scene we contemplate, while performing high-speed eye movements, called *saccades*, towards potential target positions^19^. The steps in between these saccades namely, the *fixations*, allow us to inspect the scene at high-resolution as these images fall into the central retina^20^. The fixations are therefore the events during which most visual information is captured from the target positions or from the scene^21^.

Here we address the following question: Is there any quantifiable persistent behavior in the movement of the eyes during visual search? We disclose that the process of eye movement during visual search exhibits two distinctive persistent behaviors. Initially, we condense the information about the eye paths by reducing fixations and saccades to a set of points and vectors, respectively. First, our results reveal that the probability distributions of the angles between the intersaccadic vectors present a clear asymmetry which indicates the existence of a short-range *geometrical persistence* related to a reading-like strategy of search. Second, we apply the Multifractal Detrended Fluctuation Analysis method (MF-DFA) to study the whole time series of eye movement during visual search, which encompasses fixational as well as saccadic movements. In what follows, we show that this sequential combination of distinct events leads to an apparent multifractal signature. Moreover, by inspecting the time series composed only by fixational movements, we observe that it presents instead a monofractal behavior, which is indicative of the presence of long-range power-law positive correlations. Such power-law correlations are synonymous of long-range *statistical persistence* and therefore are interpreted as a signature of long memory processes^22^.

As mentioned before, both approaches (geometrical and statistical) are not cognitive measurements, as they just arise from the analysis of the visual path. Therefore, the cognitive layer of the problem is beyond the scope of our statistical framework. However, by doing direct measurements on the eye paths we are able to address on how visual search is performed from a different perspective. In other words, we are reporting from a statistical point of view what participants do while looking for a hidden target.

## Results

### Geometrical Persistence

**Figure 1:**
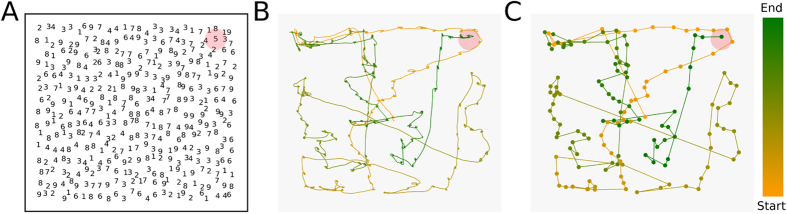
The experiment. (**A**) Visual search stimuli. Participants have to search for the unique number “5” embedded in a cloud of numbers between “1” and “9”. The red region shows the position of the target, the unique number “5”. (**B**) Eye movement recorded during the experiment. The path going from yellow to green, represents the actual eye trajectory, the color yellow corresponds to the beginning of the search and the green to the ending. (**C**) Modified eye trajectory. In order to study the eye paths as geometrical entities, we simplified the original trajectory, shown in (**B**), by reducing the fixations into points and the saccadic events into the vectors between two consecutive fixations, i.e. two points.

We study the geometrical persistence in eye movement during a visual search task by the analysis of two angles, namely, the horizontal angle, 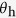, and directional angle, 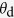. The first refers to the angle between a saccadic event and the horizontal direction, and the second corresponds to the angle between two consecutive saccadic events. The visual task consists in searching for a unique number “5” embedded in an image of numbers between “1” and “9”, as depicted in Fig. 1A. We randomly place the numbers at the nodes positions of a regular square lattice and displace them from that position by a small random distance, both in the *x* and *y* coordinates. This is done in such way that there is no overlapping between numbers (see Methods for technical details).

In order to study both the horizontal and directional angles we simplify the original trajectories, shown in Fig. 1B, by replacing the fixational and saccadic events by a series of dots and vectors, respectively. As presented in Fig. 1C, the dots correspond to the mean position of a fixation, and the vector between two fixations, i.e. two dots, represents a saccadic event (see Methods for information on the fixation and saccade filtering method adopted). By doing this we are able to study the persistence in a spatial sense, regardless the time interval of each fixation or saccade. Next, we compute 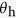 and 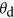. The first one provides information on the anisotropy of the trajectories, whereas the second provides information on the directional persistence.

The horizontal angle for the *i*-th saccade is computed as


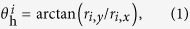


where 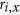 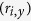 is the *x* (*y*) component of vector **r**_*i*_ associated with the *i*-th saccadic event. The range of 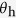 goes from 0° to 360° in a counterclockwise sense. For example, if 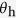 is close to 0° it means that the saccadic movement was done with a preference towards the horizontal direction from left to right. On the other hand, if 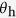 is close to 180° it means that the saccadic movement was done over the horizontal direction from right to left. The inspection of the distribution of 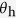 provides information of the existence of a privileged direction during the search.

**Figure 2:**
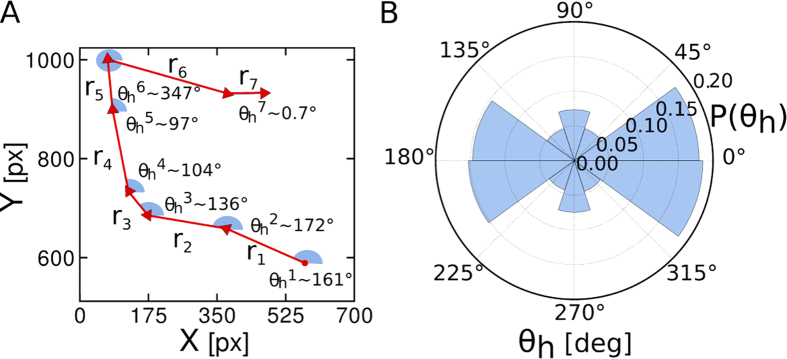
The distribution of the horizontal angle (*θ*_h_) exhibits spatial anisotropy. (**A**) Definition of the horizontal angle along the modified visual trajectory, for a real experiment. 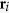 is the vector associated to a saccadic event (red arrows) as the angle 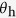 denoted in blue corresponds to the angle that goes from the horizontal direction up to 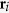 in a counterclockwise angular direction. (**B**) The distribution for 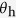 shows a clear anisotropy in space as the number of counts is larger for angles associated with the *x* gaze position. This indicates a search strategy towards a reading-like search.

Figure 2A shows schematically how 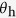 is extracted from an experimental trajectory. As one can see in Fig. 2B, the horizontal angle distribution, 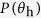, presents a clear asymmetry during the visual search. Although the images (Fig. 1A) were prepared in such a way as to prevent any salience and/or directional biases, the subjects prefer to perform a systematic search following a horizontal sweep. This is very different from a regular random walk where the subjects would perform an isotropic search with a uniform distribution for 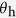, since no direction should be privileged. However, the large percentage of 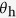
’s falling in the intervals from 315° to 45° and from 135° to 225°, between 15% and 20%, indicates a clear preference towards a reading-like search. Measuring the absolute angle relative to any other direction, rather than the horizontal one, does not change the results. For example, instead of the horizontal angle, one could measure the “vertical angle” and observe the same distribution rotated 90°.

In order to study the geometrical persistence from one saccadic movement towards the other, we defined the directional angle 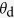, from 0° to 360°, as


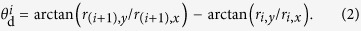


**Figure 3:**
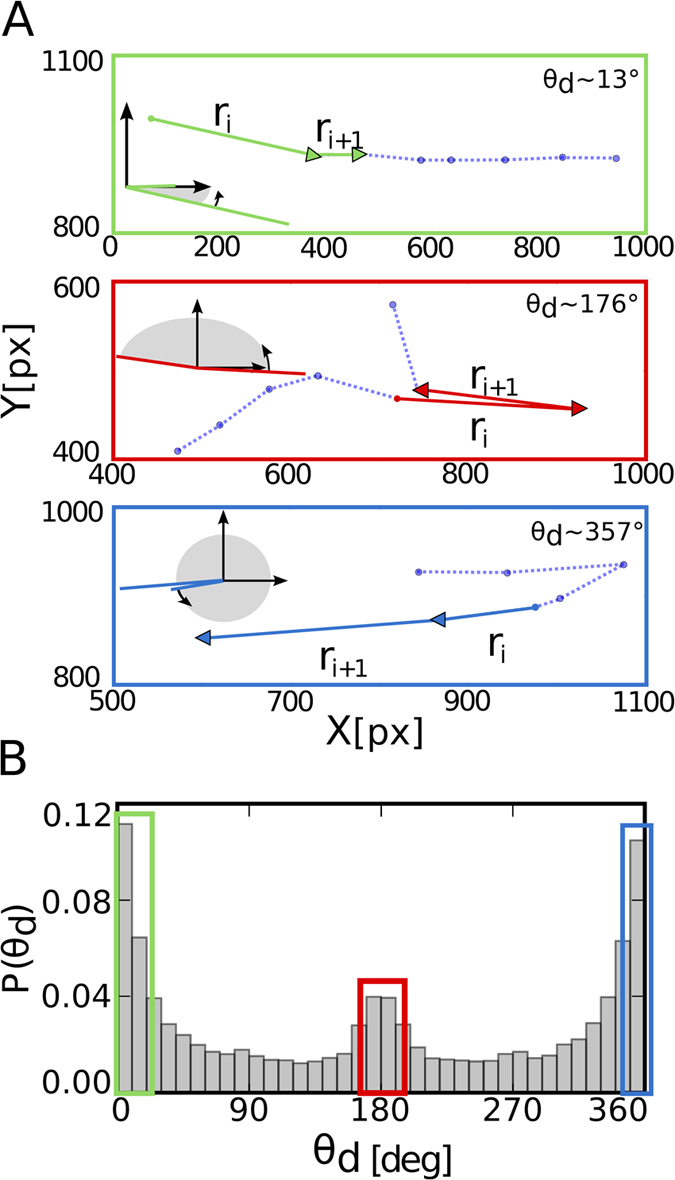
Directional persistence is unveiled by the distribution of the directional angle (*θ*_d_). (**A**) Each panel represents a different type of directional persistence. Different extracts from modified trajectories are presented along with saccade vectors 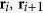 (green, red and blue arrows). The directional angle is defined as the relative angle between both vectors with an angular direction going from 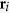 to 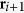 (small curved arrow). Both 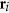 and 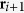 are drawn into a coordinate axes to show the angle between them, 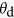 (shaded gray). From top to bottom: A movement with an almost 13° directional angle, showing counterclockwise persistence; 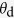 close to 180° that can be related to a turning point, or anti-persistent movement, and 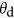 close to 360° showing clockwise persistence. (For better visualization, 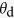 was chosen to be ≈13° instead of ≈0° to show counterclockwise persistence.) (**B**) The distribution of 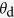 differs from a uniform distribution having large peaks at 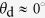 and 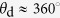, implying that persistence exists during the visual search.

Again, 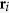 corresponds to the saccadic vector at step *i*, and 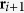 to the one at the subsequent time step *i* + 1. The quantity 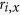 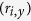 denotes the *x* (*y*) component of 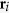 from the *i*-th saccadic event. From the definition of 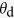, the movement will be considered persistent if consecutive saccadic jumps frequently follow a similar direction, namely, if low (close to 0°) and large (close to 360°) angle values dominate the distribution 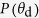. Angles with a value around 180° then correspond to anti-persistent movement, that is, a saccadic event followed by another that goes back in the opposite direction (Fig. 3A).

The distribution of 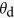, as depicted in Fig. 3B, shows a pattern that again differs from the one associated to a random searcher. If the subject performs a random search with no memory, a uniform distribution 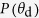 should be expected. The experimental distribution of 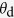 shows instead that directional persistence exists along the visual search, having a significant percentage of angles close to 0° and 360°. More precisely, 12% of the angles between saccades are close to 0°, and 10% are close to 360°. We believe that this statistics shows that the participants prefer to go on a path following a certain linear direction, at least during a short term. However, there are also some antipersitent movements (i.e. return saccades). This last is shown by the considerable number of 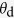 around 180°, representing 5% of all cases.

### Reading and non-reading-like search strategies

The previous results suggest that most of the participants prefer to perform a reading-like strategy while looking for the unique number “5”. This could imply that the directional persistence emerges only from those subjects performing these strategies. In other words, if a participant performs a reading like type of search, most of his saccadic movements are done sequentially on the same direction. Therefore, such trajectory exhibits a directional persistence behavior on the horizontal direction. To see if this hypothesis is valid, we separated all trials into two sets, trajectories that follow a reading-like strategy and trajectories that do not. From the histograms of 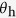 computed for each trial, we calculate the difference between the count number of angles associated to the horizontal direction and angles associated with the vertical direction. If a trial follows a reading-like type of search then this difference is very large, as there is an anisotropy towards the horizontal direction. On the other hand, this difference diminishes for a trial following a non-reading-like type of search, as there are equal contributions on the horizontal direction as well as on the vertical direction. By setting a threshold (we use a threshold equal to 0.5), we are able to divide our set of trial into two groups: trajectories that follow a reading-like type of search (81%), and trajectories that follow a non-reading-like type of search (19%).

**Figure 4:**
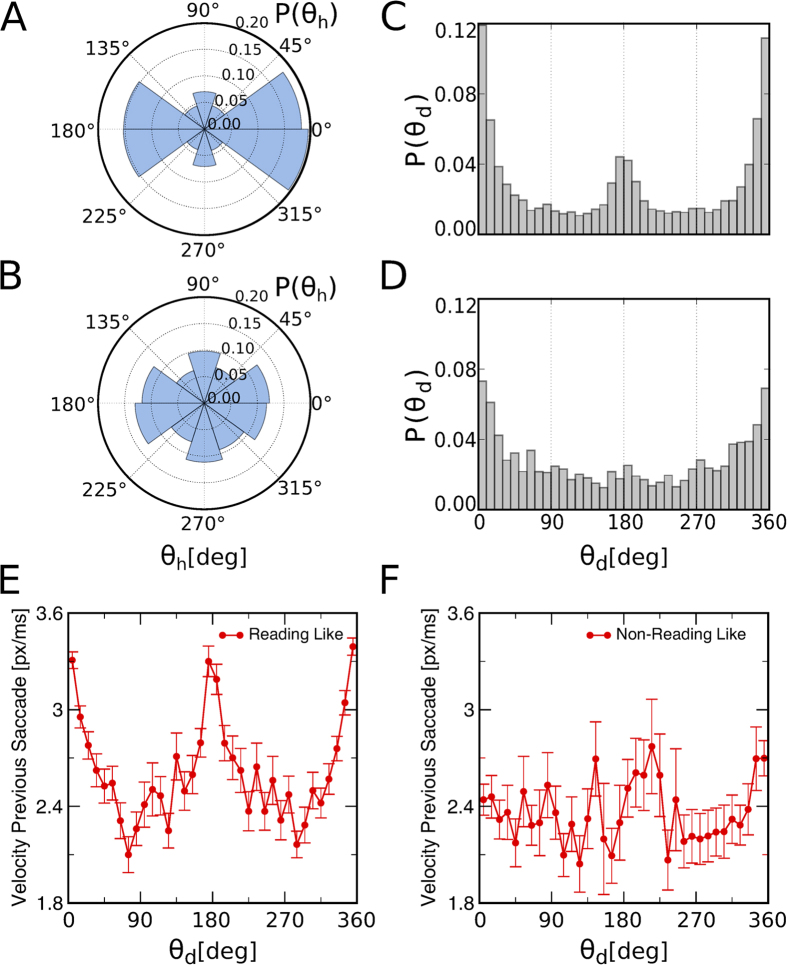
The distributions of *θ*_h_ and *θ*_d_ shows different strategies for the visual search. (**A**) Set of trials that display a reading-like trajectory for visual search. The asymmetry on the number of counts for bins between[315–45]° and [135–225]° shows a preference towards a search following the horizontal direction, namely, a reading-like search. (**B**) Set of trials that perform a type of trajectory different from a reading-like search. The distribution for 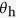 resembles more a uniform distribution. Although there is a clear asymmetry on the horizontal direction, as well as on the vertical one, it is not as prominent as the one found in (**A**). (**C**) Distribution of 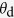 for the set of trials that follow a reading-like search. Persistent movements are revealed by the asymmetry in the distribution, with a large number of occurrences with 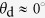 and 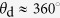. (**D**) The distribution of 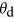 for the trials that do not show an anisotropic distribution for 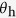, thus do not follow a reading-like strategy, also show evidence of persistent behavior on the eye movements. The asymmetry on the distribution of 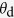, with more occurrences with 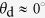 and 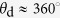, shows that persistent movements exist regardless of the strategy employed during the visual task. It is notorious, how the peak regarding antipersistence 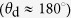 disappears and is only present in a reading-like type of trajectory. Mean velocity of the previous saccade as a function of 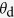 for trials following a reading-like trajectory for visual search (**E**) and for those following a type of trajectory different from a reading-like search (**F)**. Both persistent and anti-persistent saccades carried out by users enrolled on a reading-like strategy are faster than those obtained for other non-reading-like strategies. Thus, this may suggest that an automatic mechanism is at play in the case of reading-like strategies.

Trials corresponding to a reading-like strategy exhibit an asymmetry in the distribution, as expected, biased towards the horizontal direction, as shown in Fig. 4A. Those who execute a different strategy have a distribution for 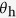 closer to a uniform one, as depicted in Fig. 4B, where the preference towards the horizontal direction diminishes. We computed the distribution of 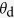 values for both groups or search strategies. Fig. 4C shows that reading-like trajectories exhibit a persistent pattern that is quite similar to the one shown in Fig. 3B for the whole set of trials. Interestingly, for the non reading-like trials directional persistence still appears, as can be seen in Fig. 4D. This reflects that directional persistence exists regardless of the selected strategy, and that 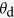 is a good indicator of that.

It is also notorious that the peak corresponding to antipersistence disappears for the non-reading-like trials. This finding needs to be studied in more detail and may be related to efficiency during visual search. As is generally accepted, in reading tasks return saccades are used by individuals to facilitate text understanding^23^,^24^. Such an automatic mechanism could be (consciously or not) used by the participants in our experiment as a way to deal with the problem of information foraging in a visual search context. This can be actually confirmed by studying the correlations between velocity and directional angles (see Figs. 4E,F). There we observe that horizontal saccades (both persistent and anti-persistent) carried out by users enrolled on a reading-like strategy are clearly faster, so suggesting that an automatic (faster) mechanism is at play. As a consequence, return saccades observed in this strategy probably correspond to revisits of the participant to a previous site that, due to the faster speed used while foraging, has not been conveniently explored. This suggests that, when using reading-like patterns, participants rely on larger saccades, so reducing the search time, in spite of the occasional delay due to return saccades.

### Short-range persistence on saccadic events

The distribution of the directional angle shows that there is a directional persistence of the eye paths treated as geometrical entities. In order to study the sequential dependence of the trajectory beyond the pair correlation between consecutive saccades, we study the conditional probability 
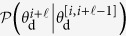
 of having a persistence movement at 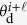 given that the previous 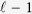 movements have also been persistent. Therefore, on a given trial, 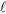 is the length of a sequence of persistent saccadic movements starting at the *i*-th element of the sequence of 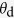. For example, imagine that a subject performs a saccadic movement at 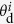. If 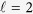, we compute the probability that 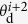 is a persistent saccadic movement given that 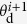 and 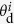 also are persistent saccadic movements. Therefore, the analysis of 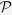 provides information on how many saccadic events a participant performs on average within the same relative direction.

**Figure 5:**
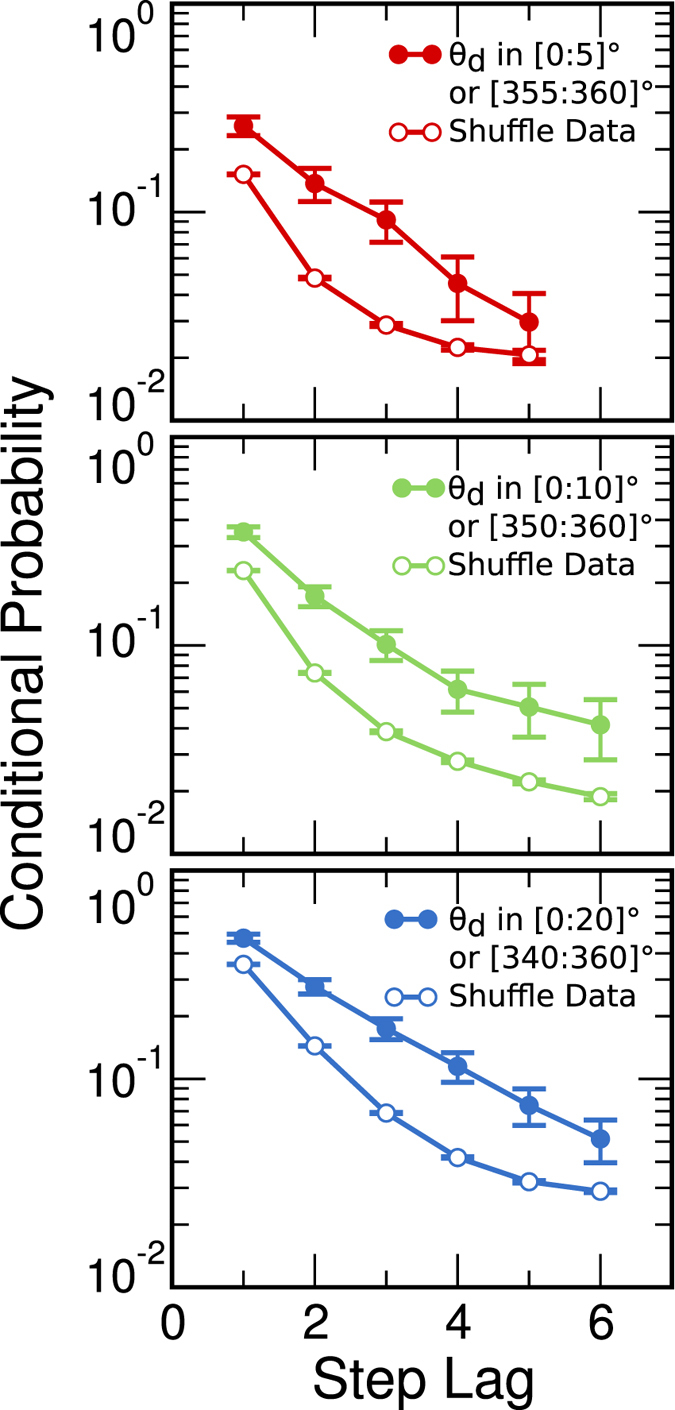
Short-term persistence on saccadic events. The conditional probability of having a value of 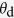 in a certain interval given that all previous 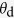
’s are also in that interval is computed for different step lags. From top to bottom: Conditional probability for 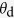 in [0:5]° or [355:360]°, for 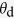 in [0:10]° or [350:360]° and for 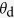 in [0:20]° or [340:360]°. The full colored dots represent the experimental data and the empty ones, the shuffled data.

We study 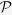 for 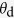 in [0:5]° or [355:360]°, which represents saccadic persistent movements in both counterclockwise and clockwise angular direction. We also study 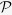 for 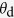 in [0:10]° or [350:360]°, and 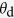 in [0:20]° or [340:360]°. Analyzing all trials we find that this sequential persistence is short-term, as shown in Fig. 5. Also shown in Fig. 5 is the dependence of 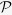 on the step lag 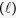 calculated after shuffling the data of the saccadic sequence. As compared to the original experiments, the faster decay of 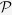 observed for the shuffled data confirms the intrinsic persistence present in the eye paths.

### Statistical Persistence

Up to here, we analyzed persistence in visual search treating the trajectories as geometrical entities. In this section, we study the long-range persistence in visual search by performing a MF-DFA^25^ over the time series of displacements magnitude in the eye trajectory involving all eye movements (see Methods for the MF-DFA method). The MF-DFA provides the multifractal spectrum identifying the *q*-order fluctuation function, 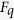, in the fractal structure within time periods with large and small fluctuations. The analysis of log-log plots of 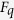 versus the scale, *s*, for each *q* value reveals the presence or not of scaling behavior in the original time series. For long-range power-law correlated signals 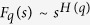, where 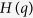 is the generalized Hurst exponent. If the profile is of the form of a fractional Brownian motion 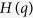 goes from 0 to 1^26^. Values of 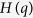 between 0 and 0.5 correspond to long-range anticorrelated time series, whereas values between 0.5 and 1 correspond to long-range positive correlated time series^27^,^28^,^29^. A Hurst exponent of 0.5 corresponds to uncorrelated time series. Multifractal time series are those that exhibit a power-law regime in 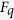 but with different 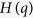 for different values of *q*, while monofractal time series are those that exhibit a power-law regime in 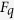 but have a constant value of 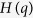 independent of *q*.

We performed the MF-DFA over the magnitude time series (also called volatility time series) which has been studied before in economics^30^,^31^, in human heartbeat fluctuations^32^, as well as in human activity^33^. For an original *x* gaze position one has a time series, *X*, of length *N* such that 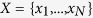. Then the magnitude time series is defined as


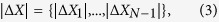


where 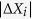 is the absolute value of the difference between two consecutive data points, 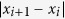.

**Figure 6:**
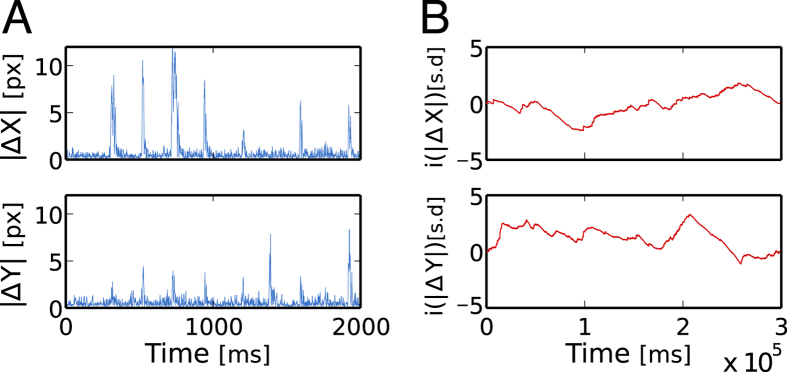
Magnitude gaze position time series and their integrated, profile, signal. (**A**) Absolute value of 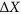

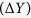
 for a typical trial time series. The actual time series is 5 minutes long, but only the first 2000 ms are shown in order to see the actual magnitude time series with enough resolution. (**B**) Integrated time series for the whole trial length showing a fractional Brownian motion nature.

The magnitude time series relates to the nonlinear properties of the original time series^32^. Assuming that the visual trajectories emerge from a complex and nonlinear underlying dynamics, studying the correlations in the magnitude time series certainly represents an adequate approach (see discussion in Supplementary information S1). An example of the magnitude time series is shown in Figs. 6A,B, for *x* and *y* position. Although the original time series is about 5 minutes long, we show only the first 2000 ms for better visualization. The integrated time series clearly evokes a fractional Brownian motion, as depicted in Figs. 6C,D.

**Figure 7:**
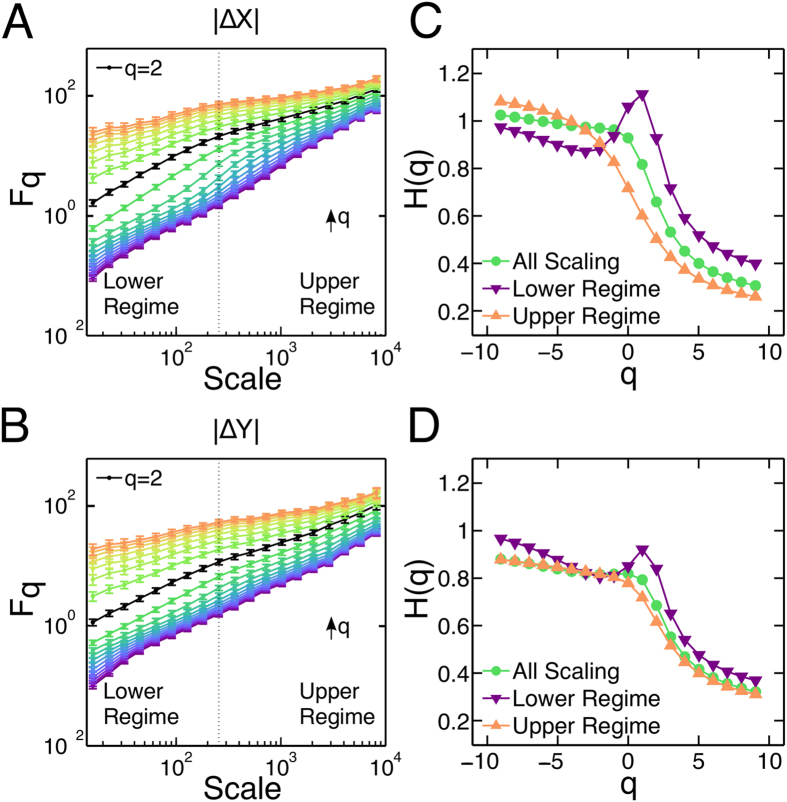
Multifractal Detrended Fluctuation Analysis (MF-DFA) of the magnitude position time series shows two scaling regimes. (**A**) and (**B)** Mean 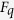 curves averaged across all trials computed for the whole magnitude position time series in *x* (**A**), and *y* (**B**). 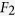 is shown in black, corresponding to the standard DFA method. A clear crossover region can be noticed at ≈300 ms. Two regimes are defined, a lower regime and a upper regime, separated by the dotted line (scale = 256 ms). The black arrow shows the direction in which the value of *q* increases, from 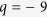 up to 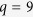. (**C**) and (**D**) Hurst exponent, *H*, as a function of *q*, for *x* (**C**) and *y* (**D**), calculated over all values (green), over the upper regime (orange) and lower regime (purple). The crossover region can be detected as the curves do not overlap for intermediate values of *q*. If 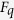 indeed would follow a power-law scaling, there should be no difference between fitting the data in one regime or another, regardless the value of *q*.

We performed the MF-DFA over all trials and then computed the mean 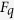 curve for each *q* value. Taking the mean 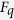 curve reduces the fluctuations across trials^34^. Next, we calculated the Hurst exponent for each 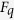 mean curve by performing a least-squares linear regression^35^. The MF-DFA of the magnitude time series involving all eye movements shows a behavior that resembles a multifractal one (Figs. 7A,B). However, there is a crossover region between 100 ms and 300 ms which suggests that the correlations do not follow a power-law scaling. This behavior is most notorious for the fluctuation functions with intermediate *q* values. Thus, we separated our scaling regime into an upper regime (*s* > 256 ms) and a lower regime (*s* < 256 ms) and computed *H* for each *q* only over the mentioned range of window sizes. The crossover becomes evident as different values of *H* are obtained for intermediate *q* values, as shown in Figs. 7C,D. The scaling exponent does not differ significantly between regimes for 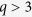 and 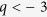. For negative values of *q*, *H* corresponds to long-range positive correlated signals being 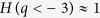 for the horizontal coordinate and 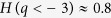 for the vertical coordinate. On the other hand, positive values of *q* correspond to a long-range anti-correlated behavior, being 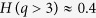 for the horizontal coordinate as well as for the vertical one. The negative values of *q* are related to small fluctuations, whereas positive *q* values are related to large fluctuations. That is, for negative *q* values, 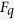 are more influenced by segments of the time series that have a small variance, while for positive *q* values, by segments with large variance. This suggests that negative *q* values are related to fixational eye movements, small and fast fluctuations, and positive *q* values to saccadic events, large fluctuations. This conjecture is reinforced by the fact that the crossover region is of the order of the average time interval of the fixations, close to 300 ms in our experiment.

Finally, note that for 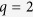 we recover the classical DFA analysis. For this case, the results in Fig. 7 coincide with those obtained from previous eye-tracking experiments performed with fixed scenes^36^,^37^ or dynamic free-viewing^38^, where a transition from positive to negative correlations has also been observed.

### MF-DFA over the fixational time series

In order to confirm the aforementioned equivalence of fixational movements to small fluctuations (and saccades to large fluctuations, correspondingly) we decided to study the time series of purely fixational movements. Each trial has a number *m* of fixational events that we extract from the original time series and ‘stitch’ together into the net fixational time series. The original time series has the form 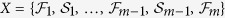, with 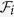 and 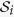 standing for the *i*-th fixation and saccade, respectively. We performed the stitching procedure by first subtracting the mean of each 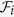, resulting in a fixation time series of the form 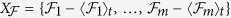. In order to avoid artificially large fluctuations, the stitching procedure is performed by joining 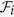 to 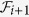 at a point where they both happen to be zero, as exemplified in Fig. S1. One could argue that the stitching procedure could emulate non-existing correlations. However, it has been shown that, given a positively correlated signal, one can remove random segments of the signal up to 50% of the total length without losing its scaling behavior^39^.

**Figure 8:**
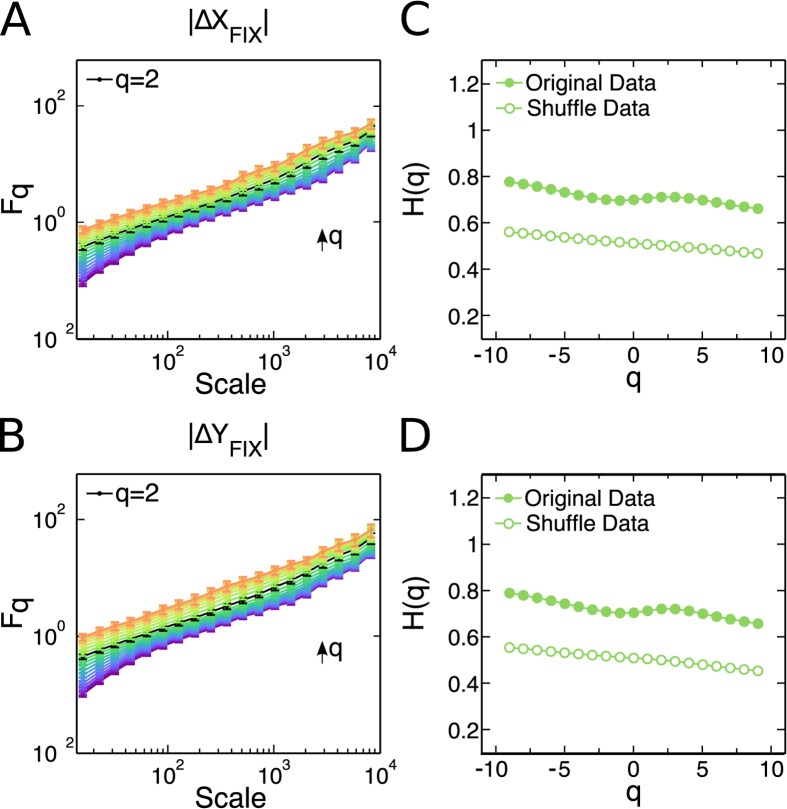
Fixation time series exhibit a long-range positive correlated monofractal behavior. (**A**) and (**B**) 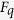 as a function of the scale computed for the fixation time series in *x* (**A**) and *y* (**B**). For all values of *q*, 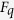 presents approximately the same slope, and it is not possible to identify two different regimes. (**C**,**D**) Hurst exponent *H* as function of *q* for the original fixation time series and the shuffled fixation time series in *x* (**C**) and in *y* (**D**). As 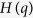 is approximately constant along all values of *q*, the fixation time series are monofractal and positively correlated with 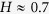.

The MF-DFA for the 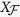 and 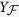 magnitude time series, for the *x* and *y* gaze directions respectively, reveals a monofractal behavior since *H* does not change along a wide range of *q* values (Fig. 8). Moreover, the obtained value 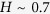 indicates that the magnitude fixation time series contains long-range positive correlations. This means that large fluctuations are more likely to be followed by large fluctuations and analogously for the small ones, indicating a possible persistent behavior on the fixational movements. By shuffling the magnitude fixation time series, we found that the correlations originates from the actual data, as 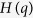 for the shuffled time series differs from the one encountered on the real data (see Supplementary information Sec. S2 for a further discussion on the shuffled data). Hence, one can conclude that the multifractal behavior of vision is necessarily a result of the combination between the saccadic and fixational movements. Interestingly, these results are robust when we remove microsaccades from the fixational events (see Supplementary information Sec. S3). The removal of microsaccadic events is performed by the conjugation of the stitching process described previously with a widely accepted microsaccade-detecting algorithm^40^.

## Discussion

We investigated the presence of two types of persistent behavior along the visual search trajectories. The first one makes reference to the trajectories as a geometrical entity, since we simplified the original trajectories replacing the fixational and saccadic events by a series of points and vectors, respectively. For this we defined two angles: 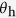, the angle between the horizontal direction and the saccade vector and 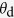, the relative angle between two saccade vectors. The distribution of 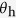 shows that most participants prefer to perform a systematic search in order to find the target, related to a reading-like search. This is revealed by the large amount of 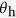
’s with values close to 0° and 180°, which indicates a spatial anisotropy. On the other hand, the distribution for 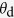 presents a bias towards values of 0° and 360°, being this related to a directional linear persistence. Altogether our analysis shows that participants are likely to adopt an automatic reading-like (either conscious or not) mechanism for searching. This is confirmed by showing that such reading-like patterns do actually correlate with faster saccades, so with faster spatial exploration. This increase in speed requires, however, the use of return saccades from time to time in order to revisit positions were some information may have been lost or overlooked. While these results seem reasonable, we stress that they are still speculative and would probably require a more rigorous verification. This could be probably attained for instance, by comparing the search strategies of literate vs illiterate subjects, in a similar fashion as conducted in ref. 41.

The second type of persistence that we study is a temporal statistical persistence over the original trajectories. By inspecting their temporal scaling, via a MF-DFA method, we found that the magnitude time series present a multifractal behavior as the fluctuation function has a different slope for each *q*-order. However, a crossover region exists that does not allow us to confirm whether or not these time series follow a power-law scaling. We believe that this is due to the fact that the time series are composed by at least two very different movements with a different underlying dynamics, one related to the fixations and the other to the saccades. When we analyze just the fixation magnitude time series, we find that these have a monofractal behavior showing long-range positive correlations and, therefore, long-range persistence over time. It is noticeable that these results are in close agreement to previous works^37^,^38^,^42^ where fixational movements (including microsaccades) were studied for other cognitive tasks. This may be interpreted as an indirect proof that saccadic mechanisms (used repeatedly during visual search) and microsaccades (typically used to compensate shorter fixational movements) share a common generator or dynamics, in agreement with recent findings^43^. This, however, would require a more complete study in which saccades and microsaccades could be compared in identical (or at least very similar) conditions.

Finally, we believe that these results should be taken into account while trying to model eye-movement during visual search, as well as in the modeling of similar visual tasks. Geometrical persistence carries information regarding the localization of the last potential target position, affecting where to go next while inspecting an image. Therefore it is not just the distribution of saccades sizes that is relevant. In fact, it appears that the statistical properties of the whole time series (persistence representing just one significant case) should be regarded as a means to uncover emergent phenomena from an underlying nonlinear system operating at a neuromotor level.

## Materials and Methods

The study has been approved by the Ethics Research Committee of the Universidade Federal do Ceará (COMEPE) under the protocol number 056/11. All methods used in this study were carried out in accordance with the approved guidelines and all experimental protocols were approved by COMEPE. Informed consent was obtained from all subjects.

## Data and Experiment

The experiment on visual search was performed using an EyeLink 1000 system (SR Research Ltd., Mississauga, Canada), with an acquisition frequency of 1 kHz on a monocular recording. Each subject carried out a sequence of four trials, each one with a maximum duration of five minutes. In each trial we presented the participant an image with numbers randomly distributed in a 1024 × 1280 px image, where the goal was to find a unique number 5. Between each trial the subject had the possibility of relaxing and before starting the recording we performed a new calibration. At the beginning of each trial the participant was asked to fixate his/her eyes into the center of the screen, in case a drift correction needed to be performed. The duration of each trial may vary, as it ends when the target is found. Ten subjects were tested from a group of graduate and undergraduate students from Universidade Federal do Ceará, who had normal or corrected to normal vision. Trials with a poor calibration or with gaze points outside the screen were disregarded.

The classification of the fixation and saccades was made using the EyeLink online filter^44^ [Section 4.3]. Fixations in the EyeLink system are identified using a saccade-pick algorithm. The system analyzes the moment-to-moment velocity and acceleration of the eye using fixed thresholds for both the velocity and acceleration of the eye. If the eye goes above either the velocity or acceleration threshold, the start of a saccade is marked. Analogously, when both the velocity and the acceleration drop back below their thresholds, the algorithm identifies the saccade end. By default, every movement which does not lie within this definition is considered as being part of a fixation. The saccade velocity threshold was set to be 30°/s, the saccade acceleration threshold, 8000°/s^2^, and the saccade motion threshold, 0.15°.

### MF-DFA method

We preform the multifractal detrended fluctuation analysis over the magnitude time series using the method described in ref. 45. First we obtained the profile 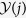 of a time series *u* of length *N* as


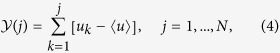


where 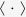 indicates the average over the whole time series. Next, the profile is divided into 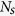 non-overlapping time windows of equal length *s*. Then, the local trend, for each window 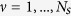 is computed by a least-squares fitting of the profile. Thus, the detrended time series for 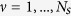 becomes,


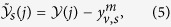


where 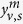 is the local polynomial trend of degree *m*. For each of the 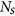 segments the variance of the detrended signal 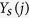 is evaluated by averaging over all data points *j* in the *v*-th window,


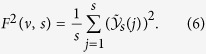


Finally, averaging over all segments *v* we obtain the *q*-order fluctuation function,


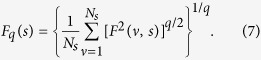


For *q* = 2 we recover the regular DFA result^28^.

## Additional Information

**How to cite this article**: Amor, T. A. *et al*. Persistence in eye movement during visual search. *Sci. Rep*. **6**, 20815; doi: 10.1038/srep20815 (2016).

## Supplementary Material

Supplementary Information

## References

[b1] Yarbus A. L. Eye movements during perception of complex objects. In Eye movements and vision (Springer, 1967).

[b2] Araujo C., Kowler E. & Pavel M. Eye movements during visual search: The costs of choosing the optimal path. Vision. Res. 41 3613–3625 (2001). 11718799 10.1016/s0042-6989(01)00196-1

[b3] Boccignone G. & Ferraro M. Modelling gaze shift as a constrained random walk. Physica A 331, 207–218 (2004).

[b4] Over E. A. B., Hooge I. T. C., Vlaskamp B. N. S. & Erkelens C. J. Coarse-to-fine eye movement strategy in visual search. Vision. Res. 47, 2272–2280 (2007). 17617434 10.1016/j.visres.2007.05.002

[b5] Rao R. P., Zelinsky G. J., Hayhoe M. M. & Ballard D. H. Eye movements in iconic visual search. Vision. Res. 42, 1447–1463 (2002). 12044751 10.1016/s0042-6989(02)00040-8

[b6] Credidio H. F., Teixeira E. N., Reis S. D. S., Moreira A. A. & Andrade J. S. Jr Statistical patterns of visual search for hidden objects. Sci. Rep. 2, 920 (2012). 23226829 10.1038/srep00920 PMC3515807

[b7] Otero-Millan J., Troncoso X. G, Macknik S. L., Serrano-Pedraza I. & Martinez-Conde S. Saccades and microsaccades during visual fixation, exploration, and search: foundations for a common saccadic generator. J. Vision 8, 21 (2008). 10.1167/8.14.21 19146322

[b8] Najemnik J. & Geisler W. S. Optimal eye movement strategies in visual search. Nature 434 ,387–391 (2005). 15772663 10.1038/nature03390

[b9] Motter B. C. & Belky E. J. The guidance of eye movements during active visual search. Vision. Res. 38, 1805–1815 ( 1998). 9797959 10.1016/s0042-6989(97)00349-0

[b10] Treisman A. M. & Gelade G. A feature-integration theory of attention. Cognitive. Psychol. 12, 97–136 (1980). 10.1016/0010-0285(80)90005-5 7351125

[b11] Palmer J. Attention in visual search: Distinguishing four causes of a set-size effect. Curr. Dir. Psychol. 4, 118–123 (1995).

[b12] Horowitz T. S. & Wolfe J. M. Visual search has no memory. Nature 394, 575–577 (1998). 9707117 10.1038/29068

[b13] Peterson M. S., Kramer A. F., Wang R. F., Irwin D. E. & McCarley J. S. Visual search has memory. Psychol. Sci. 12, 287–292 (2001). 11476094 10.1111/1467-9280.00353

[b14] da F. Costa L. Visual saliency and attention as random walks on complex networks. *preprint available at arXiv:physics/0603025v2* (2006).

[b15] Brockmann D. & Geisel T. The ecology of gaze shifts. Neurocomputing 32, 643–650 (2000).

[b16] Melcher D. Persistence of visual memory for scenes. Nature 412, 401–401 (2001). 11473303 10.1038/35086646

[b17] Melcher D. & Kowler E. Visual scene memory and the guidance of saccadic eye movements. Vision. Res. 41, 3597–3611 (2001). 11718798 10.1016/s0042-6989(01)00203-6

[b18] Melcher D. Accumulation and persistence of memory for natural scenes. J. Vision 6, 2 (2006). 10.1167/6.1.2 16489855

[b19] Liversedge S. P. & Findlay J. M. Saccadic eye movements and cognition. Trends. Cogn. Sci. 4, 6–14 (2000). 10637617 10.1016/s1364-6613(99)01418-7

[b20] Hubel D. H., Wensveen J. & Wick B. Eye, brain, and vision (Scientific American Library: New York, , 1995).

[b21] Inhoff A. W. & Radach R. Definition and computation of oculomotor measures in the study of cognitive processes (Elsevier Science Ltd, 1998).

[b22] Baillie R. T. Long memory processes and fractional integration in econometrics. J. Econometrics 73, 5–59 (1996).

[b23] Booth R. W. & Weger U. W. The function of regressions in reading: Backward eye movements allow rereading. Memory and cognition 41, 82–97 (2013). 22886737 10.3758/s13421-012-0244-y

[b24] Schotter E. R., Tran R. & Rayner K. Don’t believe what you read (only once) comprehension is supported by regressions during reading. Psychol. Sci. 25, 1218–1226 (2014). 24747167 10.1177/0956797614531148

[b25] Kantelhardt J. W. . Multifractal detrended fluctuation analysis of nonstationary time series. Physica A 316, 87–114 (2002).

[b26] Movahed M. S., Jafari G. R., Ghasemi F., Rahvar S. & Tabar M. R. R. Multifractal detrended fluctuation analysis of sunspot time series. J. Stat. Mech. Theor. Exp. 2006, P02003 (2006).

[b27] Hurst H. E. Long-term storage capacity of reservoirs. Trans. Amer. Soc. Civil. Eng. 116 (1951).

[b28] Peng C. K. . On the mosaic organization of dna sequences. Phys. Rev. E 49, 1685–1689 (1994). 10.1103/physreve.49.1685 9961383

[b29] Graves T., Gramacy R. B., Franzke C. & Watkins N. A brief history of long memory. *preprint available at arXiv:1406.6018* (2014).

[b30] Liu Y. . Statistical properties of the volatility of price fluctuations. Phys. Rev. E 60, 1390 (1999). 10.1103/physreve.60.1390 11969899

[b31] Kalisky T., Ashkenazy Y. & Havlin S. Volatility of linear and nonlinear time series. Phys. Rev. E 72, 011913 (2005). 10.1103/PhysRevE.72.011913 16090007

[b32] Ashkenazy Y. . Magnitude and sign correlations in heartbeat fluctuations. Phys. Rev. Lett. 86, 1900 (2001). 11290277 10.1103/PhysRevLett.86.1900

[b33] Hu K. . Non-random fluctuations and multi-scale dynamics regulation of human activity. Physica A 337, 307–318 (2004). 15759365 10.1016/j.physa.2004.01.042 PMC2749944

[b34] Rybski D., Buldyrev S. V., Havlin S., Liljeros F. & Makse H. A. Scaling laws of human interaction activity. Proc. Natl. Acad. Sci. USA 106, 12640–12645 (2009). 19617555 10.1073/pnas.0902667106 PMC2722366

[b35] Press W. H., Teukolsky S. A., Vetterling W. T. & Flannery B. P. Numerical Recipes in C - The art of scientific computing (Cambridge University Press, 2002).

[b36] Engbert R. & Kliegl R. Microsaccades keep the eyes balance during fixation. Psychol. Sci. 15, 431–431 (2004). 15147499 10.1111/j.0956-7976.2004.00697.x

[b37] Mergenthaler K. & Engbert R. Modeling the control of fixational eye movements with neurophysiological delays. Phys. Rev. Lett. 98, 138104 (2007). 17501244 10.1103/PhysRevLett.98.138104

[b38] Roberts J. A., Wallis G. & Breakspear M. Fixational eye movements during viewing of dynamic natural scenes. Front. Psychol. 4 (2013). 10.3389/fpsyg.2013.00797 PMC3810780 24194727

[b39] Chen Z., Ivanov P. C., Hu K. & Stanley H. E. Effect of nonstationarities on detrended fluctuation analysis. Phys. Rev. E 65, 041107 (2002). 10.1103/PhysRevE.65.041107 12005806

[b40] Engbert R. & Kliegl R. Microsaccades uncover the orientation of covert attention. Vision research. Vision Res. 43, 1035–1045 (2003). 12676246 10.1016/s0042-6989(03)00084-1

[b41] Shinde D., Mehta A. & Mishra R. K. Searching and fixating: Scale-invariance vs. characteristic timescales in attentional processes. Europhys. Lett. 94, 68001 (2011).

[b42] Moshel S. . Persistence and phase synchronisation properties of fixational eye movements. Eur. Phys. J. Special Topics 161, 207–223 (2008).

[b43] Martinez-Conde S., Otero-Millan J. & Macknik S. L. The impact of microsaccades on vision: towards a unified theory of saccadic function. Nat. Rev. Neurosci. 14, 83–96 (2013). 23329159 10.1038/nrn3405

[b44] EyeLink S. R. EyeLink 1000 User Manual (2010).

[b45] Ihlen E. A. Introduction to multifractal detrended fluctuation analysis in matlab. Front. Physiol. 3 (2012). 10.3389/fphys.2012.00141 PMC3366552 22675302

