# Supplementary material for: Persistence in eye movement during visual search
Source: Sci Rep. 2016 Feb 11;6:20815. doi: 10.1038/srep20815 (PMC4807769; doi:10.1038/srep20815)
Supplement: Supplementary Information [file srep20815-s1.pdf]

Supplementary information

Persistence in eye movement during visual search

Tatiana A. Amor,[1,2] Saulo D. S. Reis,[2] Daniel Campos,[3]
Hans J. Herrmann,[1,2] and José S. Andrade, Jr.[1,2]

[1]*Computational Physics IfB, ETH Zurich, Stefano-Franscini-Platz 3, CH-8093, Zurich, Switzerland*

[2]*Departamento de Física, Universidade Federal do Ceará, 60451-970 Fortaleza, Ceará, Brazil*

[3]*Departament de Física, Universitat Autònoma de Barcelona, 08193 Bellaterra, Barcelona, Spain*

(Dated: October 29, 2015)

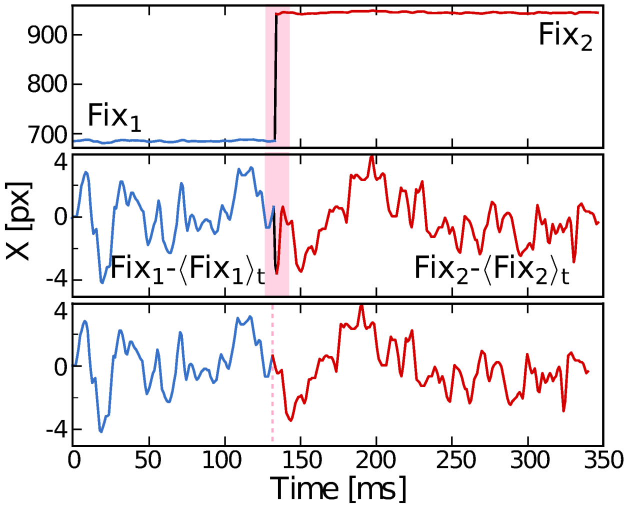

Figure S1. *Stitching process for the fixation time series.* From top to bottom: Two fixational events are identified in the whole trial time series, Fix_1 (red) and Fix_2 (blue). After subtracting the mean from each fixation, they are concatenated together showing an artificial large fluctuation that does not belong to the actual data (black line on pink shaded region in middle panel). By removing data points from the fixation, such that they start and end crossing the zero value, the artificial fluctuation is removed and the two fixations are now stitched.

S1: MAGNITUDE TIME SERIES

The analysis of temporal persistence was performed over the magnitude time series as they give an insight on the nonlinearities. In order to see if this approach is correct, we performed the MF-DFA over the increment time series. The increment time series, for an original gaze time series X, is of the form,

$$\Delta X = \{\Delta X_1, ..., \Delta X_{N-1}\}, \tag{1}$$

where ΔX_i is the difference between two consecutive data points, $x_{i+1} - x_i$. Different from the magnitude time series, the increment time series carries not only the magnitude of the increment but also the sign.

In Fig. S2A and B we show the MF-DFA over the whole trials increment time series, that is, involving all ocular movements. For positive and large q values, the curves corresponding to the fluctuation function do not differ from the ones obtained for the magnitude series

(Figs. 9A and B). However, when inspecting the curves corresponding to negative q values, the behavior differs from the one found for the magnitude time series.

When performing MF-DFA over the increment fixational time series we found that for most q values the scaling corresponds to a Hurst exponent of 0, indicating the presence of long-range anticorrelations on the time series (Figs. S2C and D). Even more, the fluctuation function corresponding to positive q values shows a perfect scaling with an exponent equal to zero. On the other hand, the fluctuation found for negative values of q and large values of s exhibits the same behavior as the one found for positive q values. For small s the fluctuation function for negative q values has a different trend that correlates to the one found on the magnitude fixational time series (see Figs.10A and B).

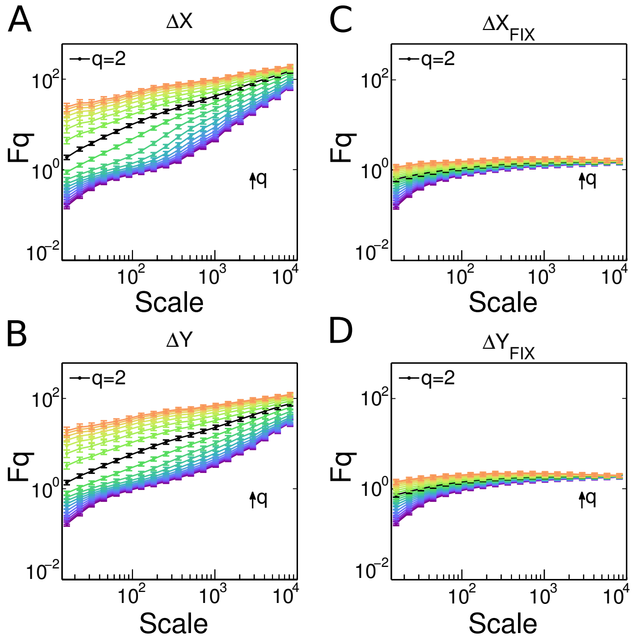

Figure S2. *MF-DFA over the increment time series.* **(A)** and **(B)** MF-DFA performed over the increment time series for the whole trial, that is, involving all eye movements. **(C)** and **(D)** MF-DFA over the increment fixational time series. The fluctuation functions found for the increment fixational time series have a Hurst exponent $H \sim 0$, corresponding to anticorrelated time series. This change in scaling from the increment time series to the magnitude time series reinforces the hypothesis that the analysis of the magnitude time series reveals the nonlinearities of the visual system.

The fact of having different scaling values for the magnitude time series and the increment time series was thoroughly studied on heartbeat fluctuation time series [31]. When studying

the increment time series, linear and nonlinear properties are in play, resulting in scaling exponents corresponding to anticorrelated signals. By taking the magnitude time series and performing a detrended fluctuation analysis, the scaling exponent changes, suggesting that the magnitude time series carries information regarding the nonlinear properties of the heartbeat dynamics. Having inspected the increment time series for our experiment, and given that the results found relate to the ones found on the time series for heartbeat fluctuations, the study on the magnitude time series gives an insight on the visual dynamics.

S2: MAGNITUDE DISTRIBUTION IN THE FIXATIONAL TIME SERIES

In Section III.A we investigated the presence of long-range correlations on the magnitude fixational time series. When performing the MF-DFA over the shuffled data, we found that although it differs from the result found for the real data, the curve $H(q)$ vs q does not follow a straight line on 0.5 for the Hurst exponent (as expected for uncorrelated time series). In order to understand this result we generate a set of artificial time series that have the same distribution of the magnitude time series and perform MF-DFA over this set. We generated a set of artificial time series by applying a rejection method sampling (RM) using the distribution for the magnitude fixational time series shown in Fig. S3. The rejection method is based on a simple geometrical argument. Given a known distribution $P(x)$, one selects values from a uniform distribution both for x and $P(x)$, then if the sorted values fall inside the area under the curve defined by $P(x)$, that sample is accepted, otherwise it is rejected. (For further reading on RM we refer to Ref.[34, Section 7.3].)

The set of artificial time series, which we will refer as RM time series, has by construction the property of exhibiting a specific distribution but without temporal correlations. Making this analysis allows us to differentiate between temporal correlations existing in our data and some spurious correlations associated with the distribution itself. Fig. S4 shows $H(q)$ for the already presented results both for the original data and the shuffled data, as well as the results obtained for the RM series. There is no evident statistical difference between the points obtained by shuffling the original data and the ones from the RM series. Even more, the behavior found in the shuffled data can also be observed on the RM series, implying that it is due to the distribution of the magnitude time series. Our analysis therefore suggests that the long-range correlations found with MF-DFA over the magnitude fixational time

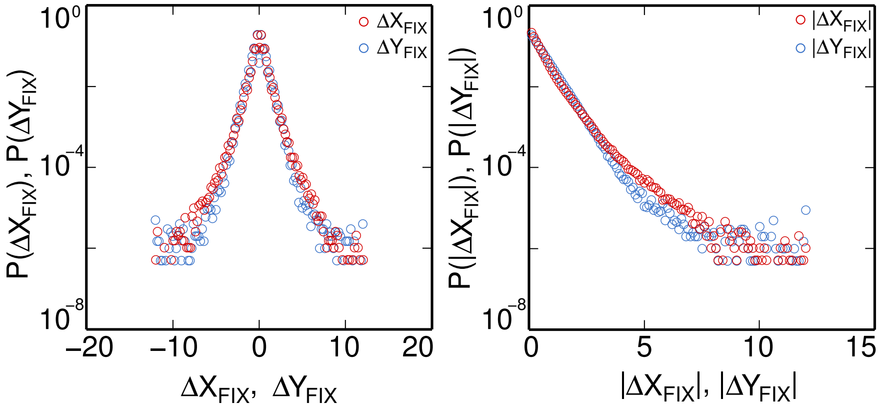

Figure S3. *Distribution of values of the fixational time series.* The left panel shows the distribution for the increment time series of the x coordinate (red) and y coordinate (blue). On the right panel it is the distribution of the magnitude time series, for the horizontal and vertical coordinates.

series are due to the data itself, and not having a straight line on 0.5 for $H(q)$ on the shuffled data is a consequence of the distribution of magnitude values.

S3: MICROSACCADIC MOVEMENTS IN THE MAGNITUDE FIXATIONAL TIME SERIES

In order to see whether the results found in Section III.A for the magnitude fixational time series are robust without the presence of microsaccadic events, we implemented the detection algorithm of microsaccades developed by Engbert and Kliegl [40]. We applied this algorithm on the experimental fixations and used the stitching procedure, as described in section III.A., to remove detected microsaccadic events. In the implementation of the detection algorithm we set the minimum time duration of a microsaccadic movement to be 12ms, as described in Ref.[40]. After that, we performed the MF-DFA method over the fixational time series removing the microsacadic events and we found that the Hurst exponent differs from the one presented in the manuscript by $\approx 1\%$. As shown in Fig. S5, the MF-DFA analysis of the fixational time series after deleting the microsaccades presents no significant change while comparing to the original results presented in Figs. 8C and 8D of the manuscript.

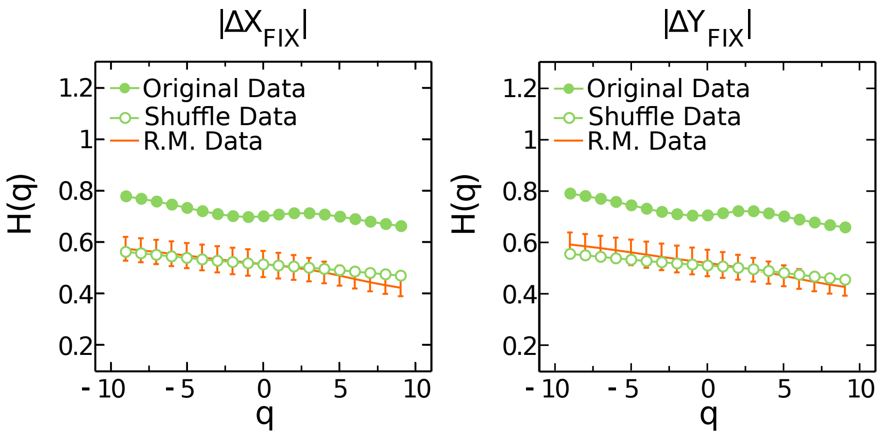

Figure S4. *MF-DFA results for the rejection method sampling time series set.* Hurst exponent as a function of q for the magnitude fixational time series (full green dot), and two different shuffling methods for the horizontal coordinate (left panel) and vertical coordinate (right panel). The results obtained shuffling the original data (white face dots) does not follow a straight line centered in a H value of 0.5(uncorrelated time series). This result is due to the type of distribution from the fixational magnitude values. Such a behavior is confirmed by generating a set of artificial time series by applying a rejection method sampling (RM data) that, by construction, preserves the distribution of the original data set but with no temporal correlations. $H(q)$ for the R.M data (orange) has the same behavior as the one found for the shuffling of the original data.

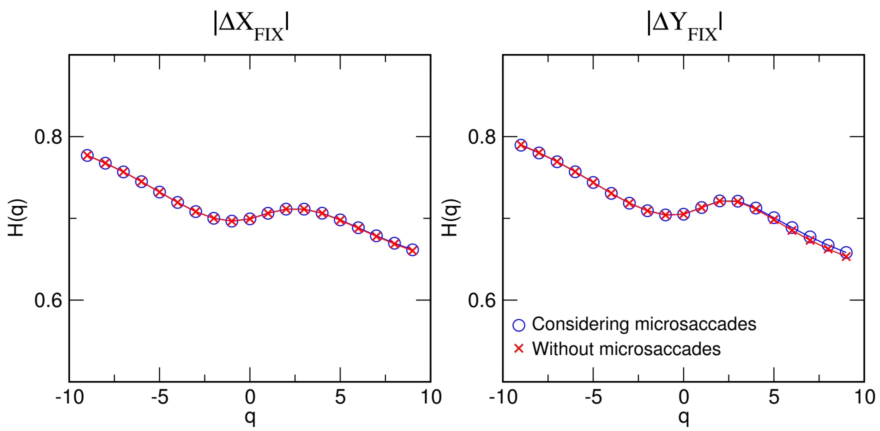

Figure S5. Hurst exponent, H, as a function of q for the magnitude fixation time series over the x and y position. The results considering microsaccades (blue circles) do not present significant differences to the ones obtained by identifying and removing the microsaccades (red crosses).
